# Acute and sub-chronic toxicological evaluation of the herbal product HAD-B1 in Beagle dogs

**DOI:** 10.1016/j.toxrep.2021.11.002

**Published:** 2021-11-05

**Authors:** Si-Yeon Song, Hyeok-Joon Chang, Soo-Dam Kim, Eun-Bin Kwag, So-Jung Park, Hwa-Seung Yoo

**Affiliations:** East West Cancer Center, Daejeon Korean Medicine Hospital, Daejeon University, 75, 176 Bun-Gil, Daedeok-daero, Seo-gu, Daejeon City, 35-235, Republic of Korea

**Keywords:** Acute toxicity, Sub-chronic study, Lung cancer, Korean traditional medicine, Herbal product, HAD-B1, No observed adverse effect level

## Abstract

HAD-B1 is a herbal formula originated from Korean Traditional Medicine that used to treat lung cancer patients. Herein we assessed acute and sub-chronic toxicity of HAD-B1 in beagle dogs. Acute study, 4 weeks dose rate finding (DRF) study and sub chronic toxicity study for 13 weeks were done by oral administration at doses of 0, 500, 1000, and 2000 mg/kg. Neither oral acute toxicity study nor DRF study showed any significant clinical signs, death, or weight changes. Based on that, a sub-chronic study for 13-weeks was performed. As a result, HAD-B1 caused a decrease of mean daily feed consumption in females, infiltration of intestinal inflammatory cells in both sexes, a significant decrease in total cholesterol (TCHO) in females, Kupffer cell hypertrophy/hyperplasia in the liver as well as dilation of the sinusoid. However, there were no significant toxic effects in the treated group compared to the control group. Therefore, the No Observed Adverse Effect Level (NOAEL) of the HAD-B1 is at least 2000 mg/kg/day when administrated orally for 13 consecutive weeks. These results demonstrate that HAD-B1 consumption is relatively non-toxic and safe for clinical usage.

## Highlights

• HAD-B1 is used in Korean medicine to treat lung cancer patients.
• The acute and Sub-chronic toxicity of HAD-B1 was evaluated in Beagle dogs.
• No toxicologically significant effects were found.
• The NOAEL was ≥2000 mg/kg/day for both genders, with no target organ effect.

## 1 Introduction

According to World Health Organization (WHO), 65∼80 % of the world’s population have traditional medicines as part of their healthcare system [1]. One of the oldest forms of traditional medicine is herbal medicine [2]. As the usage of herbal medicine increased, more scientific evidence regarding the safety of herbal products are in great demands [3]. Even though traditional herbal formulas are generally thought to be safe to use, toxicology evaluations are in shortfall [[4], [5], [6], [7]]. Therefore, adequate safety assessments are needed to certify the continuous use of herbal plants for medical purposes and phytotherapy [[7], [8], [9]].

HAD-B1 comprises four kinds of herbs which are *Panax ginseng* C.A.Mey, *Panaxnotoginseng* Radix, *Cordyceps militaris*, *Boswellia carterii* BIRDWOOD. Herbs are extracted into liquid form at a low temperature of 60℃, freeze-dried, and given to patients in tablet form. Previously, anti-tumor effects of HAD-B1 have been reported. It significantly inhibited solid tumor growth in nude mice bearing an A549 human lung cancer xenograft [10]. The anti-carcinogenic properties of these four substances are as follows. It has been found that ginseng’s saponin components affect lung cancer cells’ apoptosis (A549, H23, H1299, lung squamous cell carcinoma cell lines et al) [[11], [12], [13]] and stimulate immune cells such as Th1/Th2 helper cells [14]. Ginsenoside Rg1, Rg3, and other components found in *Panax ginseng* are known to affect the mechanism of lung cancer cell apoptosis [[11], [12], [13]]. *Panaxnotoginseng*’s saponin components also control lung cancer cells’ apoptosis and showe anti-carcinogenic properties during animal testing [[15], [16], [17]]. *Cordyceps militaris* can control the mechanism of non-small-cell lung cancer cells as well as the mechanism of apoptosis in other lung cancer cells [[18], [19], [20]]. *Boswellia carterii* BIRDWOOD’s anti-carcinogenic mechanisms are slowly coming into the light, and the combination of these four substances are expected to block the various possible progressions of lung cancer cells’ apoptosis as well as preventing further metastasis [21].

In this study, we planned to investigate oral toxicity, acute effect, time-dependent chronic effects, and safety in the medicinal use of HAD-B1 with this background knowledge. Based on the previous study, the no observed adverse effect level (NOAEL) of HAD-B1 for rats was >2000 mg/kg based on the acute and sub chronic toxicological evaluation [22]. Accordingly, this present study aims to further investigate to assess the NOAEL of HAD-B1 in beagle dogs through an acute oral toxicity study, 4-weeks repeated oral DRF study, and a 13-weeks repeated sub-chronic oral dose toxicity and 4-weeks recovery study.

## 2 Materials and methods

### 2.1 Overall design of the toxicology program

The animal study for acute oral toxicity was conducted by Korea Testing and Research Institute (KTR), Hwasun., Hwasun-gun, Jeollanam-do, Republic of Korea. The 4-weeks repeated DRF toxicity study and 13-weeks repeated dose toxicity and 4-weeks recovery study was conducted at Nonclinical Research Institute, ChemOn Inc., Yongin-si, Gyeonggi-do, Republic of Korea. These studies complied with the nonclinical test management standards from the Ministry of Food and Drug Safety (MFDS) and the Organization for Economic Cooperation and Development Principle of Good Laboratory Practice (OECD), No. 409.

Acute toxicity study research protocol was reviewed and assessed (IAC2015-0120, IAC2015-0418, IAC2015-1231) following the regulation for the Institutional Animal Care and Use Committee (IACUC) at KTR, Hwasun based on Animal Protection Act [Enforcement Date 2015-01-20] [No. 13023 (Jan20,20215, partial revision)] and Laboratory Animal Act [Enforcement Date 2013-07-30] [No. 11987 (July30, 2013, partial revision)]. Protocols for the 4-week DRF study and 13-week dose toxicity study assessed by the ChemOn Icn. Animal Experimentation Ethics Committee. Nonclinical Research Institute (Review number: 19-D599), Association for Assessment and Accreditation of Laboratory Animal Care (AAALAC) accredited institution in 2010.

### 2.2 Materials

**Fig. 1:**
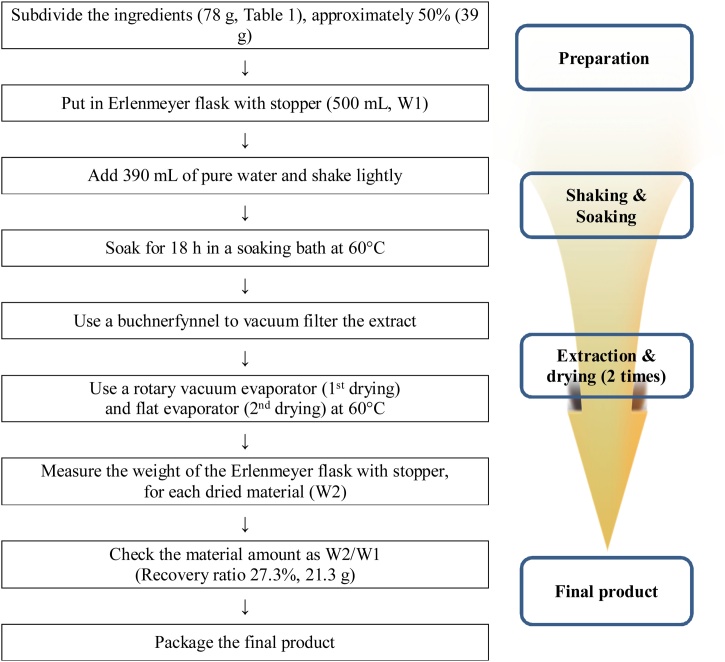
Process of HAD-B1 extraction.

HAD-B1 was provided by Kyoung Bang pharmaceutical Co. (Namdong-daero, Namdong-gu, Incheon Metropolitan city, Korea) (Table 1). The ingredients of HAD-B1 were steeped in a water for 18 h at 60 °C. After the fluid extracts had been dried twice with a rotary vacuum evaporator and a flat evaporator at 60 ℃, the powder (recovery ratio: 27.3 %) was gained for the experiment (Fig. 1).

**Table 1:** Ingredients of HAD-B1.

Scientific Name	Relative Amount (g)
*PanaxNotoginseng Radix*	*25.2*
*Cordycepsmilitaris*	*19.2*
*Panax ginseng C.A.Mey.*	*19.2*
*Boswellia carterii BIRDWOOD*	*14.4*
Total amount	78.0

### 2.3 Experimental animals and housing conditions

Beagles were provided from Beijing Marshall Biotechnology Co., Ltd. (Wayag Village, Liucun Town, Chang Ping District, Beijing 102204, China), and supplied by Woojungbio., Co. Ltd. (145, Gwanggyo-ro, Yeongtong-gu, Suwon-si, Gyeonggi-do, Republic of Korea).

24 (12 males and 12 females) beagle dogs were used in the oral acute toxicity study, aged 6 months and weighed 6.60–7.42 kg (males)/6.01–7.42 kg (females) at the beginning of dose administration. The DRF study used 5 males and 5 female dogs, they were between 5.5–6.5 months old. At the initial dose administration, body weights ranged from 7.02-7.58 kg for males and 5.76-6.86 kg for females. For a 13-week repeated oral toxicity study, 34 (17 males and 17 females) dogs were brought in. However, the actual numbers used were 32 (16 males, 16 females). The beagle dogs for the 13-week study were 6–7 months of age and weighed 6.58–8.46 kg (males)/5.06–6.96 kg (females). The rest of the dogs that were not assigned in to the treatment group were returned to the original site.

Pre-test periods were different for each trial, it was 29 days for oral acute toxicity study, 15 days for 4-weeks study, and 19 days for a 13-week study. During all pre-test periods, weights of the animals were measured weekly and they were observed daily for the overall condition. In addition, blood tests (hematological and biochemical) were performed and only healthy animals were used for the test.

During the experiment, the animals were identified by the numbers tattooed on the auricle. Individual identification cards of different colors were attached to the breeding box. The identification records were attached to the door of each animal accommodation.

The temperature of the room was maintained at 23 ± 5℃ with a relative humidity of 50 ± 20 %. The air recycling rate of 10–20 times/h and the light intensity was 150–300 Lux, with a 12-h light/dark cycle. Throughout the entire study period, temperature and relative humidity were monitored automatically every half-hour by automatic instruments, and the environmental conditions were measured periodically. The animals were fed 300 g/day of a pelleted diet [Cargill Co., Ltd. (Hallymwon Building, Bundang-gu, Seongnam-si, Gyeonggi-do, Korea), and 5LL9 (PMI, USA)], and given reverse osmosis water ad libitum in individual stainless-steel cages.

### 2.4 Dose preparation

For each study, proportion and content of HAD-B1 was kept the same, however dosage of the product was changed each time based on each animal’s most recent weight. In the acute toxicity study, the test substance, HAD-B1, was dissolved and suspended in distilled water (Daihan Pharm Co., Ltd, Seoul, Korea). In DRF and 13 weeks studies, HAD-B1 loaded to gelatin capsules and was administrated orally. HAD-B1 capsules were manufactured every 3 days and pre-made capsules were stored in the same condition and were taken out on the day of the administration. On weight measurement day, HAD-B1 was manufactured and administered on the same day.

### 2.5 Study of acute oral toxicity

Total of twelve dogs were divided into 4 groups. Group 1 was a control group, Group 2 is 500 mg/kg BW, Group 3 is 1000 mg/kg BW, and Group 4 is 2000 mg/kg BW (3 dogs/sex/group). Highest does was determined to be 2000 mg/kg following limit dose guideline. Dosage for middle and low-dose groups was reduced by half from the highest dosage (1000 and 500 mg/kg), with a volume of 5 mL/kg BW. The study was performed by oral gavage using a stomach tube (sonde) and no food was given to the dogs for approximately 18 h prior to dosing. The control group was administered with distilled water.

#### 2.5.1 Clinical observation and examination

In acute oral toxicity study, each beagle was monitored for toxicity signs and behavioral changes at 0.5, 1, 2, 3, and 4 h after the test substance administration and once daily thereafter for 14 days. Body weights were recorded before the administration, on days 7, 14, and at termination. On day14, all survived beagle dogs were euthanatized and necropsies were performed, then any gross observations were recorded.

### 2.6 Study of DRF(4-week) and Sub-chronic toxicity (13-week)

HAD-B1 was administered once daily orally by gelatin capsules at doses of 0, 250, 500, 1000, or 2000 mg/kg/day for the 4-week DRF study. In the sub chronic toxicity study, Beagle dogs were divided into 5 groups of control, 500 mg/kg/day, 1000 mg/kg/day, 2000 mg/kg/day groups. 2 beagle dogs (1 male and 1 female) were assigned into each groups. Animals in the control group were given empty gelatin capsules at the same times when the treatment group was administered with HAD B-1. After all studies has concluded, 2 males and 2 females dogs from the control group and highest dose group were observed for a recovery period of additional 4 weeks.

#### 2.6.1 General observation

During the experiment, any types of abnormal symptoms including death were observed once a day and documented for each animal. Body weight was monitored on the start of administration, once a week, upon necropsy. Food supplied and food remained were both recorded and the differences were calculated and recorded.

#### 2.6.2 Ophthalmic examination

Animal eyes were observed before the experiments start and 2 weeks after the administration, and lastly 1 week before the autopsy. As a result of observation, no abnormal symptoms of the eyeball were observed, so fundus examination was not performed. The animals were assessed for any ocular abnormalities with a fundus camera (Genesis-D, Kowa Co. Ltd., Japan) and a direct ophthalmoscope. Pupils were dilated by mydriatica [Midriasil (Lot No. 18IC03CC), Alcon Korea]. The observations were performed in the 4-week study during pretest, week 2 of treatment, within 1 week of the planned autopsy, and in the 13-week study during pretest, week 4 of treatment, within 1 week of the planned autopsy. No abnormal symptoms of the eyeball were observed in the 4-week study, so fundus examination was not performed. In 13 weeks of repeated study, a picture of the right eyeball was taken in each animal in the control and highest dose group before autopsy. No abnormal symptoms were observed, so a photograph was not taken of the left eyeball.

#### 2.6.3 Electrocardiograms

In the 4-week study, electrocardiograms were recorded from all the animals during pretest, week 2 of treatment and within 1 week of planned autopsy. In the 13-week study, electrocardiograms recordings were obtained once during pretest, and weeks 4 and within 1 week of planned autopsy. The QRS period, QT interval, and heart rate were measured by using an ECG tester (Cardiofax Vet, NIHON KOGDEN) for all animals.

#### 2.6.4 Urinalysis

In the 4-week study, urinalysis was performed by taking about 0.3 mL of fresh urine collected using a urine collection plate for all animals before administration, 2 weeks after administration, and within 1 week before scheduled autopsy. For the 13-week study 1 mL of fresh urine samples were collected at pretest, weeks 4 of treatment and within 1 week of planned autopsy. The urine volume was measured by collecting urine continuously for 24 h.The urinalysis was performed to inspect the appearance, volume, specific gravity, protein, glucose, pH, ketone body, urobilinogen, bilirubin, nitrite, blood, leukocyte, urinary sediments. An automatic tester (CliniTek 500, Siemens, USA), urine stick (Multistix 10 SG, Siemens, USA), and a microscope (Leica, Germany) were used to perform the analysis.

#### 2.6.5 Hematology and clinical biochemistry

Animals were fasted overnight (16−24 hours) but water intake was allowed prior to blood collection. Blood samples were drawn from the lumbar cortical vein before and after administration and at week 2 and on the day of autopsy in a 4-week study. In the 13-week study, blood samples were collected at pretest, week 4 of treatment, and autopsy data. The hematology parameters measured were the following: white blood cell count (WBC), differential count (neutrophils, lymphocytes, monocytes, basophils), red blood cell count (RBC), hemoglobin concentration (HGB), Hematocrit (HCT), mean corpuscular volume (MCV), mean corpuscular hemoglobin (MCH), mean corpuscular hemoglobin concentration (MCHC), red blood cell distribution width (RDW), hemoglobin distribution width (HDW), platelets (PLT), prothrombin time (PT), mean platelet volume (MPV), and activated partial thromboplastin time (APTT).

A broad range of biochemistry including aspartate aminotransferase (AST), alanine aminotransferase (ALT), alkaline phosphatase (ALP), gamma-glutamyl transferase (GGT), total bilirubin (TBIL), glucose (GLU), creatine phosphate activating enzyme (CPK), total cholesterol (TCHO), triglycerides (TG), total protein (TP), albumin (ALB), albumin/globulin ratio (A/G), blood urea nitrogen (BUN), creatinine kinase (CRE), inorganic phosphorus (IP), calcium (Ca2+), sodium (Na+), potassium (K+), and chloride (Cl-) were measured.

#### 2.6.6 Necropsy

At the end of the studies, all animals were killed and a necropsy was performed. After collecting blood for clinical pathology examination, anesthesia is performed by intravenous administration using pentobarbital (Entoval, Hallym Pharmaceutical), and then euthanized by cutting off the Axillary Artery & Vein, followed by observing and recording all organs of the somatic, oral, head, chest cavity and abdominal cavity.

#### 2.6.7 Organ weight measurement

During the autopsy, the following organs of all animals were weighed with an electronic scale (ED3202S-CW, Sartorius AG, Germany), and the relative weight of each organ to the body weight was calculated. Organs that were measured are ovary, uterus, adrenal gland, pituitary gland, thymus, prostate gland, testis, epididymis, spleen, kidney, heart, lung, brain, liver with the gallbladder, thyroid gland with parathyroid gland. Paired organs were measured respectively.

#### 2.6.8 Preservation of tissues and organs

Organs of all animals and gross abnormal organs observed at autopsy were removed, and all organs except for the eyes (Davidson's solution), testes, and epididymis (Bouin's solution) were fixed in 10 % neutral buffered formalin solution.

#### 2.6.9 Histopathological examination

All organ and tissue samples for which gross findings were recognized collected at necropsy from the 13-week study were made into tissue slides. Histopathological findings were inputted into the Provantis® (INSTEM, UK) program and processed, and diagnostic terms were mainly referred on the Standardized System for Nomenclature and Diagnostic Criteria-Guides for Toxicologic Pathology, INSTEM's Provantis Glossary and INHAND published by the American Society of Toxic Pathology. Other Covance glossary and pathology books were also selected for reference. In general, the grading was performed from +1 for minimal lesions to +5 for massive lesions. For the 4-week DRF study histopathology was not processed.

### 2.7 Statistical analysis

No statistical analysis was conducted on the data from the 4-week DRF study because only one pair of animals were used per group, therefore there was not enough data. Statistical analysis used ANOVA & Dunnt's in the ProvantisTM 10.10.1 package. However, parametric multiple comparison procedures were used for comparison between administration groups on the electrocardiogram. For statistical analysis, SPSS Statistics 22 for Medical Science, a widely used statistical package, was used. The significance difference of the electrocardiogram was evaluated by One-way ANOVA test. Level of significance was considered at values of *P* < 0.05. The two arms of the recovery group were analyzed by the Student *t*-test. Unless otherwise noted, 'significant' means that it has statistical significance compared with the control group.

## 3 Results

### 3.1 Study of acute oral toxicity

There were no deaths or any relevant clinical signs and body weight change at any dose group. (Table 2). In addition, no gross lesions were observed in any of the organs upon necropsy. Based on these acute toxicity test results, the approximate lethal dose (ALD) of HAD-B1 was considered to be over 2000 mg/kg BW for both sexes of beagle dogs. Therefore, dosage levels of 500, 1000, and 2000 mg/kg BW were selected for the DRF study of sub-chronic toxicity.

**Table 2:** Body weight of acute oral toxicity.

Group	Dose (mg/kg BW)	Sex	N	Days after administration		
				0	7	14
G1	0	Male	3	7.085 ± 0.444	7.301 ± 0.457	7.615 ± 0.377
		Female	3	6.818 ± 0.550	6.971 ± 0.562	7.256 ± 0.494
G2	500	Male	3	6.922 ± 0.278	7.154 ± 0.340	7.390 ± 0.233
		Female	3	6.858 ± 0.564	6.979 ± 0.528	7.236 ± 0.478
G3	1000	Male	3	7.189 ± 0.218	7.352 ± 0.303	7.588 ± 0.200
		Female	3	6.561 ± 0.483	6.713 ± 0.487	7.003 ± 0.200
G4	2000	Male	3	7.133 ± 0.322	7.293 ± 0.348	7.505 ± 0.313
		Female	3	6.670 ± 0.564	6.865 ± 0.527	7.138 ± 0.524

N: number of animals.

### 3.2 4-Week DRF study

#### 3.2.1 General observation

There were no abnormal clinical signs recorded regarding the test substance. Food leftover was sporadically observed on days 2–28 in the entire test group of females in group of 500 mg/kg/day or more, and the control group of males. Soft stools were irregularly observed on days 2–23 in males in group of 250, 500 mg/kg/day, and at days 8–29 in females in group of 500 and 2000 mg/kg/day. Diarrhea was observed on day 24 in males in group of 2000 mg/kg/day and in females at 1000 and 2000 mg/kg/day. Foamy vomiting was observed on days 10–11 in female control group and on day 7 in males in group 250 mg/kg/day. The vomiting of food was recorded on day 27 in males in group 500 mg/kg/day, also vomiting of test capsules was observed on day 3 in females in group 2000 mg/kg/day. These changes were observed without dose-dependency and common reactions in experiments with beagle dogs. In addition, in the case of vomiting of test article, the development of vomiting in dogs may be subjected to mild gastrointestinal stimulation without toxicity and given that it occurred only once, it was determined that it was not from the test substance. Also, there were no significant differences in body weight related to HAD-B1.

#### 3.2.2 Body weight and food consumption

Regarding body weight and food consumption, no statistically significant differences related to the test article was observed. Although food consumption declined in males in 500, 1000, 2000 mg/kg/day and 1000, 2000 mg/kg/day for females, no dose or time-response correlation has been observed.

#### 3.2.3 Ophthalmic examination, electrocardiograms, urinalysis, hematology and biochemistry

During the study period, no changes related to the administration of the test material were observed as a result of the ophthalmic examination, ECG urinalysis, hematology, and biochemistry (data not shown).

#### 3.2.4 Necropsy findings and organ weights

No abnormal findings from the test material were observed in any organs except the thin right horn of the uterus was observed without dose-dependency in females in group 500 mg/kg/day. The absolute and relative weight of the spleen showed a tendency to incline in males in group 500 mg/kg/day compared to the controls but declined in females in group 1000 mg/kg/day (Table 8).

### 3.3 13-Week repeated dose toxicity study

#### 3.3.1 General observation

No mortality occurred during the experiment and recovery periods. Food leftover was sporadically observed on days 1–92 in the control and entire test groups. Vomiting was recorded on days 10–91 in the control and the entire test groups. Soft stool and diarrhea were irregularly observed on days 3–93 in all groups. Diarrhea was observed on day 24 in males in group 2000 mg/kg/day and in females in group 1000 and 2000 mg/kg/day. Salivation and licking chops were observed at day 32–46 and day 32–33, respectively, in males of the control group.

Food leftover was sporadically observed in 1 male in 2000 mg/kg/day (day 93–94) and 1 case in the control group (day 99–119), and 2 cases in group 2000 mg/kg/day (day 94–119) in females. Vomiting of food was observed sporadically between day 101–118 in 1 male of the control group and one female in group 2000 mg/kg/day during the recovery period. The soft stool was observed between day 92–96 in one male and female in the control group and 1 male and 2 female dogs at 2000 mg/kg/day. Diarrhea was observed between day 93–118 in females in 1 case of the control group and 2 cases in group 2000 mg/kg/day.

#### 3.3.2 Body weight

Changes in body weight related to the test substance were not observed during the experiment. A significant decline in body weight was found in males in group 2000 mg/kg/day during the recovery period (*P* < 0.01).

#### 3.3.3 Food consumption

The reduction in mean food consumption in female was observed in group 1000 mg/kg/day on day 35, 70 (*P* < 0.05), and in group 2000 mg/kg/day on day 7, 14, 35, 49, 70 (*P* < 0.05, *P* < 0.01). During the recovery period, no significant affect was observed caused by the test substance.

#### 3.3.4 Ophthalmic examination

There were no effects on the ophthalmic examination related to HAD-B1, during the pretest, test, and the recovery period.

#### 3.3.5 Electrocardiograms

No treatment-related electrocardiograms were found during the study period. The duration of QRS in females in group 2000 mg/kg/day was significantly reduced during the recovery period (*P* < 0.05, data not shown).

#### 3.3.6 Urinalysis

No effects considered related to treatment with the test substance at pretest and recovery period. The urinary volume was remarkably reduced in females at 1000 mg/kg/day on week 4 (*P* < 0.05) and in males in group 1000 mg/kg/day on week 13 (*P* < 0.05).

#### 3.3.7 Hematology and biochemistry

Differences related to the test item before treatment were not recorded. HGB increased or decreased significantly in males at 500 and 1000 mg/kg/day, respectively (*P* < 0.05). HCT (%) was remarkably increased in males at 500 mg/kg/day (*P* < 0.05). APTT was notably reduced in males at 1000 mg/kg/day (*P* < 0.05). (Table 3) (Table 4)

**Table 3:** Thirteen-weeks repeated dose toxicity: hematological values of male dogs (main study) and hematological values of female dogs (main study).

Summary of hematological tests (sex: male)				
	Main group (mg/kg/day)			
	G1 (0)	G2 (500)	G3 (1000)	G4 (2000)
RBC (^6/μL)	7.19 ± 0.50 (5)	7.76 ± 0.29 (3)	6.44 ± 0.32 (3)	6.95 ± 0.52 (5)
HGB (g/dL)	16.24 ± 0.84 (5)	17.77* ± 0.35 (3)	14.8* ± 0.5 (3)	15.8 ± 0.83 (5)
HCT (%)	49.16 ± 3.23 (5)	54.6* ± 1.11 (3)	45.63 ± 2.01 (3)	48.22 ± 3.09 (5)
MCV (fL)	68.36 ± 0.9 (5)	70.33 ± 1.4 (3)	70.83 ± 1.82 (3)	69.38 ± 1.11 (5)
MCH (pg)	22.62 ± 0.66 (5)	22.87 ± 0.55 (3)	23 ± 0.78 (3)	22.76 ± 0.68 (5)
MCHC (g/dL)	33.08 ± 0.68 (5)	32.5 ± 0.66 (3)	32.47 ± 0.4 (3)	32.76 ± 0.53 (5)
RDW (%)	1.14 ± 0.21 (5)	1.11 ± 0.18 (3)	0.94 ± 0.62 (3)	1.17 ± 0.42 (5)
HDW (g/dL)	12.86 ± 0.34 (5)	12.17 ± 0.45 (3)	12.43 ± 0.21 (3)	12.58 ± 0.5 (5)
RET (%)	1.56 ± 0.08 (5)	1.487 ± 0.074 (3)	1.487 ± 0.122 (3)	1.494 ± 0.074 (5)
PLT (^3/μL)	267.4 ± 50.6 (5)	259.3 ± 15.4 (3)	278.3 ± 75.1 (3)	342.2 ± 75.3 (5)
MPV (fL)	9.94 ± 0.63 (5)	9.7 ± 0.72 (3)	11.07 ± 1.67 (3)	9.88 ± 0.88 (5)
WBC (^3/μL)	9 ± 0.72 (5)	10 ± 2.05 (3)	9.12 ± 0.22 (3)	10.16 ± 1.90 (5)
NEU (%)	58.18 ± 1.73 (5)	57.23 ± 3.75 (3)	51.83 ± 6.4 (3)	53.72 ± 11.5 (5)
LYP (%)	32.38 ± 2.07 (5)	33.57 ± 4.83 (3)	36.5 ± 4.64 (3)	36.02 ± 8.93 (5)
MONO (%)	5.54 ± 1.12 (5)	5.93 ± 2.14 (3)	6.5 ± 0.62 (3)	6.66 ± 2.61 (5)
EOS (%)	2.92 ± 0.96 (5)	2.27 ± 0.55 (3)	3.83 ± 0.67 (3)	2.42 ± 0.75 (5)
BASO (%)	0.66 ± 0.35 (5)	0.67 ± 0.21 (3)	0.73 ± 0.4 (3)	0.68 ± 0.28 (5)
LUC (%)	0.32 ± 0.18 (5)	0.3 ± 0.1 (3)	0.57 ± 0.4 (3)	0.48 ± 0.26 (5)
PT (Seconds)	5.54 ± 0.4 (5)	5.73 ± 0.6 (3)	5.8 ± 0.79 (3)	5.4 ± 0.29 (5)
APTT (Seconds)	13.84 ± 0.71 (5)	13.93 ± 0.6 (3)	12.67* ± 0.12 (3)	13 ± 0.39 (5)

Summary of hematological tests (sex: female)				
	Main group (mg/kg/day)			
	G1 (0)	G2 (500)	G3 (1000)	G4 (2000)
RBC (^6/μL)	7.35 ± 0.72 (5)	7.54 ± 0.62(3)	7.59 ± 0.68(3)	6.79 ± 0.87(5)
HGB (g/dL)	16.72 ± 1.81 (5)	16.77 ± 1.82 (3)	16.9 ± 0.89 (3)	15.54 ± 2.21 (5)
HCT (%)	51.28 ± 5.85 (5)	50.7 ± 4.71 (3)	51.47 ± 3.02 (3)	47.62 ± 6.62 (5)
MCV (fL)	69.78 ± 2.69 (5)	67.13 ± 1.16 (3)	67.93 ± 3.76 (3)	70.08 ± 1.4 (5)
MCH (pg)	22.78 ± 0.7 (5)	22.2 ± 0.87 (3)	22.37 ± 1.66 (3)	22.89 ± 0.57 (5)
MCHC (g/dL)	32.64 ± 0.54 (5)	33.07 ± 0.67 (3)	33.07 ± 0.67 (3)	32.62 ± 0.22 (5)
RDW (%)	1.11 ± 0.23 (5)	0.72 ± 0.27 (3)	0.85 ± 0.29 (3)	0.68 ± 0.34 (5)
HDW (g/dL)	12.6 ± 0.4 (5)	12.77 ± 0.4 (5)	12.07 ± 0.35 (3)	12.4 ± 0.64 (5)
RET (%)	1.46 ± 0.06 (5)	1.48 ± 0.03 (3)	1.49 ± 0.07 (3)	1.46 ± 0.13 (5)
PLT (^3/μL)	229.2 ± 82.8 (5)	279.3 ± 38 (3)	283.3 ± 36.7 (3)	907.8 ± 75.2 (5)
MPV (fL)	10.42 ± 1.63 (5)	9.7 ± 0.26 (3)	9.27 ± 0.49 (3)	10.66 ± 1.18 (5)
WBC (^3/μL)	9.85 ± 1.9 (5)	8.45 ± 2.02 (3)	7.7 ± 1.66 (3)	9.22 ± 1.39 (5)
NEU (%)	55.52 ± 7.75 (5)	58.3 ± 6.97 (3)	56.77 ± 7.32 (3)	57.92 ± 4.39 (5)
LYP (%)	31.64 ± 2.63 (5)	33.2 ± 5.27 (3)	34.57 ± 4.77 (3)	33.64 ± 3.98 (5)
MONO (%)	5.6 ± 1.51 (5)	5.07 ± 1.16 (3)	5.2 ± 1.99 (3)	5.62 ± 0.69 (5)
EOS (%)	6.12 ± 5.94 (5)	0.42 ± 0.05 (3)	2.2 ± 1.04 (3)	1.56 ± 0.51 (5)
BASO (%)	0.68 ± 0.25 (5)	2.2 ± 1.04 (3)	0.7 ± 0.26 (3)	0.68 ± 0.48 (5)
LUC (%)	0.36 ± 0.15 (5)	0.18 ± 0.08 (3)	0.5 ± 0.26 (3)	0.64 ± 0.18 (5)
PT (Seconds)	5.16 ± 0.35 (5)	5.27 ± 0.15 (3)	5.27 ± 0.15 (3)	5.32 ± 0.32 (5)
APTT (Seconds)	13.78 ± 1.64 (5)	14.23 ± 0.55 (3)	14.23 ± 0.55 (3)	13.36 ± 1.04 (5)

Mean ± SD (Number of animals). Significant difference compared with the control group value: *P < 0.05.

RBC: Red blood cell, HGB: Hemoglobin, HCT: Hematocrit, MCV: Mean corpuscular volume, MCH: Mean corpuscular hemoglobin, MCHC: Mean corpuscular hemoglobin con- centration, RDW: Red cell distribution width, HDW: Hemoglobin distribution width, RET: Reticulocyte, PLT: Platelet, MPV: Mean platelet volume, WBC: White blood cell, NEU: Neutrophil, LYP: Lymphocyte, MONO: Monocyte, EOS: Eosinophil, BASO: Basophil, LUC: Large unstained cell, PT: Prothrombin time, APTT: Activated partial thromboplastin time.

**Table 4:** Thirteen-weeks repeated dose toxicity: biochemical values of male dogs (main study) and serum biochemical values of female dogs (main study).

Summary of biochemical tests (sex: male)				
	Main group (mg/kg/day)			
	G1 (0)	G2 (500)	G3 (1000)	G4 (2000)
AST (IU/L)	34 ± 3.01 (5)	38.23 ± 2.06 (3)	40.33 ± 9.94 (3)	45.34 ± 8.09 (5)
ALT (IU/L)	35.98 ± 6.23 (5)	48.87 ± 6.54 (3)	33.07 ± 5.78 (3)	47.78 ± 10.63 (5)
ALP (IU/L)	90.5 ± 26.4 (5)	97.83 ± 4.9 (3)	120.1 ± 22.24 (3)	156.76 ± 58.56 (5)
GGT (IU/L)	2.27 ± 0.29 (5)	1.77 ± 0.5 (3)	2.32 ± 0.61 (3)	2.3 ± 0.33 (5)
CPK (IU/L)	216 ± 25.8 (5)	302.7 ± 152 (3)	318 ± 125.9 (3)	332.6 ± 138.3 (5)
TBIL (㎎/dL)	0.19 ± 0.03 (5)	0.22 ± 0.03 (3)	0.22 ± 0.02 (3)	0.22 ± 0.03 (5)
GLU (㎎/dL)	101.64 ± 6.42 (5)	96.7 ± 10.54 (3)	97.07 ± 5.38 (3)	91.84 ± 10.05 (5)
TCHO (㎎/dL)	159.8 ± 28.9 (5)	162 ± 52.4 (3)	133 ± 7 (3)	126.4 ± 13.4 (5)
TG (㎎/dL)	37.8 ± 7 (5)	52.7*±5 (3)	39 ± 5.6 (3)	32.4 ± 4.6 (5)
TP (g/dL)	5.9 ± 0.31 (5)	5.96 ± 0.15 (3)	5.86 ± 0.22 (3)	5.89 ± 0.28 (5)
ALB (g/dL)	2.86 ± 0.11 (5)	2.97 ± 0.09 (3)	2.87 ± 0.05 (3)	2.83 ± 0.16 (5)
A/G ratio	0.94 ± 0.06 (5)	0.99 ± 0.06 (3)	0.97 ± 0.08 (3)	0.92 ± 0.04 (5)
BUN (㎎/dL)	16.2 ± 2.25 (5)	17.27 ± 0.59 (3)	18.8 ± 1.54 (3)	17.1 ± 3.31 (5)
CREA (㎎/dL)	0.6 ± 0.07 (5)	0.72 ± 0.12 (3)	0.67 ± 0.07 (3)	0.72 ± 0.04 (5)
IP (㎎/dL)	5.83 ± 0.87 (5)	4.87 ± 0.96 (3)	5.87 ± 0.14 (3)	5.26 ± 0.7 (5)
Ca (㎎/dL)	10.72 ± 0.13 (5)	10.61 ± 0.08 (3)	10.5 ± 0.45 (3)	10.61 ± 0.13 (5)
Na (㎜/L)	145.36 ± 1.78 (5)	142.99 ± 0.38 (3)	144.33 ± 2.54 (3)	143.92 ± 1.03 (5)
K (㎜/L)	4.58 ± 0.18 (5)	4.64 ± 0.15 (3)	4.59 ± 0.3 (3)	4.61 ± 0.23 (5)
Cl (㎜/L)	111.57 ± 2.06 (5)	109.32 ± 0.67 (3)	109.93 ± 1.81 (3)	109.3 ± 1.33 (5)

Summary of biochemical tests (sex: female)				
	Main group (mg/kg/day)			
	G1 (0)	G2 (500)	G3 (1000)	G4 (2000)
AST (IU/L)	34.06 ± 7.18 (5)	45.7 ± 11.44 (3)	39.67 ± 11.12 (3)	42.32 ± 8.16 (5)
ALT (IU/L)	32.36 ± 1.86 (5)	37.8 ± 4.33 (3)	55.1 ± 24.57 (3)	45.06 ± 28.66 (5)
ALP (IU/L)	113.26 ± 27.6 (5)	128.6 ± 20.9 (3)	147.57 ± 62 (3)	122.24 ± 57.15 (5)
GGT (IU/L)	1.68 ± 0.69 (5)	1.93 ± 0.79 (3)	1.82 ± 0.97 (3)	2 ± 0.39 (5)
CPK (IU/L)	224.6 ± 45.7 (5)	286.7 ± 23 (3)	104 ± 249.4 (3)	249.4 ± 60 (5)
TBIL (㎎/dL)	0.21 ± 0.02 (5)	0.26 ± 0.08 (3)	0.24 ± 0.02 (3)	0.22 ± 0.02 (5)
GLU (㎎/dL)	100.14 ± 7.95 (5)	93.07 ± 5.95 (3)	85.07*±8.75 (3)	91.32 ± 3.3 (5)
TCHO (㎎/dL)	194.6 ± 36.4 (5)	161 ± 28.9 (3)	141.3 ± 29.6 (3)	123.2**±24.2 (5)
TG (㎎/dL)	42.8 ± 10.9 (5)	55.3 ± 17.6 (3)	47.7 ± 17 (3)	34.8 ± 8.8 (5)
TP (g/dL)	6.01 ± 0.16 (5)	5.98 ± 0.38 (3)	5.91 ± 0.22 (3)	5.77 ± 0.38 (5)
ALB (g/dL)	2.84 ± 0.26 (5)	2.83 ± 0.17 (3)	2.89 ± 0.07 (3)	2.95 ± 0.17 (5)
A/G ratio	0.91 ± 0.17 (5)	0.9 ± 0.08 (3)	0.96 ± 0.08 (3)	1.05 ± 0.11 (5)
BUN (㎎/dL)	14.94 ± 2.44 (5)	19.43 ± 1.54 (3)	19.13 ± 2.55 (3)	17.08 ± 2.52 (5)
CREA (㎎/dL)	0.64 ± 0.04 (5)	0.76 ± 0.05 (3)	0.74 ± 0.05 (3)	0.93***±0.14 (5)
IP (㎎/dL)	4.9 ± 0.3 (5)	4.8 ± 0.55 (3)	4.79 ± 0.31 (3)	4.91 ± 0.82 (5)
Ca (㎎/dL)	10.8 ± 0.23 (5)	10.58 ± 0.09 (3)	10.69 ± 0.38 (3)	10.86 ± 0.12 (5)
Na (㎜/L)	143.56 ± 1.53 (5)	141.24 ± 4.26 (3)	144.32 ± 0.5 (3)	143.21 ± 0.88 (5)
K (㎜/L)	4.23 ± 0.12 (5)	4.38 ± 0.39 (3)	4.46 ± 0.2 (3)	4.6 ± 0.28 (5)
Cl (㎜/L)	110.51 ± 0.5 (5)	108.72 ± 3.69 (3)	110.64 ± 0.96 (3)	109.71 ± 1.2 (5)

Mean ± SD (Number of animals). Significant difference compared with the control group value: *P < 0.05; **P < 0.01; ***P < 0.001.

AST: Aspartate aminotransferase, ALT: Alanine aminotransferase, ALP: Alkaline phosphatase, GGT: Gamma glutamyl transpeptidase, CPK: Creatine phosphokinase, TBIL: Total bilirubin, GLU: Glucose, TCHO: Total cholesterol, TG: Triglyceride, TP: Total protein, ALB: Albumin, A/G ratio: Albumin/Globulin ratio, BUN: Blood urea nitrogen, CREA: Creatinine, IP: Inorganic Phosphorus, Ca: Calcium ion, Na: Sodium ion, K: Potassium ion, Cl: Chloride Ion.

Before administration, ALP was notably increased in males at 500 mg/kg/day group and males at 2000 mg/kg/day AST and CPK (*P* < 0.05, *P* < 0.01). In week 4, TG was notably increased in males at 500 mg/kg/day and BUN in females at 500 and 1000 mg/kg/day (*P* < 0.05, *P* < 0.01), and Cl- in females at 500 and 2000 mg/kg/day and female at 1000 mg/kg/day showed a notably decline in K+ (*P* < 0.05). TG was significantly high in males at 500 mg/kg/day, CRE was high in females at 2000 mg/kg/day (*P* < 0.05, *P* < 0.001) in week 13. GLU in females at 1000 mg/kg/day and TCHO in females at 2000 mg/kg/day were notably low (*P* < 0.05, *P* < 0.01). During recovery, the A/G ratio significantly reduced in males at 2000 mg/kg/day (*P* < 0.05). AST and CPK significantly decreased in females at 2000 mg/kg/day (*P* < 0.05, *P* < 0.01), and K+ and Cl- increased remarkably in females at 2000 mg/kg/day (*P* < 0.05).

#### 3.3.8 Organ weights

The results of the organ weight (Table 5) are as follows: No relevant differences were recorded related to treatment in control and experimental groups. However, in recovery group, right testis, left thyroid and parathyroid gland relative weight, stomach absolute weight, right thyroid and parathyroid gland absolute and relative weight were increased in males in group 2000 mg/kg/day (*P* < 0.05). The relative weights of the right testicles, left thyroid and parathyroid glands, the absolute weights of gastric, the absolute and relative weight of right thyroid and parathyroid glands, were significantly increased in males in group 2000 mg/kg/day (*P* < 0.05). Females in group 2000 mg/kg/day, the absolute right adrenal weight decreased significantly (*P* < 0.05), and the relative weight of the pituitary gland significantly increased (*P* < 0.05).

**Table 5:** Thirteen-weeks repeated dose toxicity: absolute and relative organ weights of male dogs (main study) and absolute and relative organ weights of female dogs (main study).

Summary of absolute organ weights				
	Group (mg/kg/day) sex: male			
	G1 (0)	G2 (500)	G3 (1000)	G4 (2000)
Body weight (kg)	9.013 ± 0.902 (3)	8.893 ± 1.163 (3)	9.607 ± 1.122 (3)	9.127 ± 1.47 (3)
Adrenal gland_L (g)	0.59 ± 0.082 (3)	0.597 ± 0.125 (3)	0.59 ± 0.053 (3)	0.7 ± 0.135 (3)
% Adrenal gland_L (petcent)	0.00664 ± 0.00155 (3)	0.00669 ± 0.0079 (3)	0.00615 ± 0.00018 (3)	0.00779 ± 0.00196 (3)
Adrenal gland_R (g)	0.603 ± 0.058 (3)	0.573 ± 0.167 (3)	0.593 ± 0.059 (3)	0.73 ± 0.154 (3)
% Adrenal gland_R (percent)	0.00679 ± 0.0014 (3)	0.00643 ± 0.00148 (3)	0.00619 ± 0.00019 (3)	0.00807 ± 0.0019 (3)
Pituitary gland (g)	0.05 ± 0.01 (3)	0.05 ± 0.01 (3)	0.057 ± 0.012 (3)	0.067 ± 0.021 (3)
% Pituitary gland (percent)	0.00055 ± 0.00006 (3)	0.000057 ± 0.00012 (3)	0.00006 ± 0.00017 (3)	0.00075 ± 0.00028 (3)
Thymus	10.203 ± 7.06 (3)	9.7 ± 3.726 (3)	7.847 ± 1.035 (3)	9.183 ± 1.055 (3)
% Thymus (percent)	0.10946 ± 0.07202 (3)	0.10708 ± 0.03339 (3)	0.08231 ± 0.01403 (3)	0.10109 ± 0.00434 (3)
Prostate gland (g)	3.867 ± 0.177 (3)	2.75 ± 1.254 (3)	4.06 ± 1.126 (3)	3.603 ± 3 (3)
% prostate gland (percent)	0.04349 ± 0.01107 (3)	0.03013 ± 0.01029 (3)	0.04346 ± 0.01578 (3)	0.03707 ± 0.02564 (3)
Testis_L (g)	6.763 ± 1.056 (3)	5.76 ± 1.625 (3)	5.253 ± 0.559 (3)	5.127 ± 1.599 (3)
% Testis_L (percent)	0.07622 ± 0.01925 (3)	0.06386 ± 0.01077 (3)	0.05508 ± 0.00789 (3)	0.05571 ± 0.01246 (3)
Testis_R (g)	6.38 ± 0.823 (3)	5.693 ± 1.145 (3)	5.237 ± 0.807 (3)	5.073 ± 1.179 (3)
% Testis_R (percent)	0.07162 ± 0.01483 (3)	0.06361 ± 0.00473 (3)	0.05508 ± 0.01145 (3)	0.05571 ± 0.01131 (3)
Epididymis_L (g)	1.323 ± 0.345 (3)	0.953 ± 0.23 (3)	1.057 ± 0.0238 (3)	1.093 ± 0.315 (3)
% Epididymis_L (percent)	0.01453 ± 0.00255 (3)	0.01061 ± 0.00127 (3)	0.01105 ± 0.00268 (3)	0.01189 ± 0.00224 (3)
Epididymis_R (g)	1.223 ± 0.246 (3)	0.99 ± 0.105 (3)	0.108 ± 0.111 (3)	1.103 ± 0.0313 (3)
% Epididymis_R (percent)	0.01348 ± 0.00147 (3)	0.01116 ± 0.00034 (3)	0.01132 ± 0.00156 (3)	0.01198 ± 0.00191 (3)
Spleen (g)	36.22 ± 13.21 (3)	35.347 ± 2.256 (3)	27.787 ± 3.115 (3)	32.773 ± 9.376 (3)
% Spleen (percent)	0.40201 ± 0.135 (3)	0.40065 ± 0.04128 (3)	0.28941 ± 0.000911 (3)	0.36859 ± 0.13962 (3)
Kidney_L (g)	20.083 ± 1.175 (3)	18.243 ± 2.209 (3)	21.19 ± 1.604 (3)	20.467 ± 2.212 (3)
% Kidney_L (percent)	0.22356 ± 0.01219 (3)	0.20737 ± 0.00381 (3)	0.22128 ± 0.01014 (3)	0.22594 ± 0.02258 (3)
Kidney_R (g)	19.853 ± 1.362 (3)	18.157 ± 2.867 (3)	20.98 ± 2.438 (3)	21.147 ± 3.835 (3)
% Kidney_R (percent)	0.22078 ± 0.00832 (3)	0.20365 ± 0.00598 (3)	0.21864 ± 0.01392 (3)	0.23168 ± 0.02 (3)
Heart (g)	71.92 ± 3.81 (3)	69.21 ± 8.935 (3)	78.197 ± 8.608 (3)	72.083 ± 6.4 (3)
% Heart (percent)	0.8006 ± 0.04022 (3)	0.77854 ± 0.01527 (3)	0.81882 ± 0.10967 (3)	0.7956 ± 0.05499 (3)
Lung (g)	78.053 ± 3.098 (3)	76.79 ± 8.162 (3)	82.087 ± 16.141 (3)	77.853 ± 4.646 (3)
% Lung (percent)	78.78 ± 0.295 (3)	75.257 ± 8.063 (3)	75.73 ± 6.52 (3)	80.167 ± 3.538 (3)
Brain (g)	0.88004 ± 0.09002 (3)	0.849 ± 0.0491 (3)	0.79026 ± 0.03165 (3)	0.89611 ± 0.16642 (3)
% Brain (percent)	256.937 ± 26.306 (3)	200.38 ± 32.709 (3)	246.55 ± 39.17 (3)	223.19 ± 16.79 (3)
Liver&gall bladder (g)	256.937 ± 26.306 (3)	2.25361 ± 0.20139 (3)	2.56616 ± 0.2954 (3)	2.48377 ± 0.041348 (3)
% Liver&gall bladder (percent)	2.85576 ± 0.2022 (3)	0.427 ± 0.067 (3)	0.417 ± 0.13 (3)	0.413 ± 0.289 (3)
Thyroid gland + PG_L (g)	0.487 ± 0.153 (3)	0.427 ± 0.067 (3)	0.417 ± 0.13 (3)	0.413 ± 0.289 (3)
% Thyroid gland + PG_L (percent)	0.00533 ± 0.00129 (3)	0.00479 ± 0.00029 (3)	0.00446 ± 0.00176 (3)	0.0043 ± 0.00233 (3)
Thyroid gland + PG_R (g)	0.443 ± 0.035 (3)	0.433 ± 0.102 (3)	0.419 ± 0.136 (3)	0.377 ± 0.168 (3)
% Thyroid gland + PG_R (percent)	0.00493 ± 0.00027 (3)	0.00485 ± 0.00066 (3)	0.0053 ± 0.0019 (3)	0.00401 ± 0.00109 (3)

Summary of absolute organ weights				
	Group (mg/kg/day) sex: female			
	G1 (0)	G2 (500)	G3 (1000)	G4 (2000)
Body weight (kg)	7.427 ± 0.961 (3)	7.02 ± 0.644 (3)	6.413 ± 0.34 (3)	6.847 ± 0.67 (3)
Ovary_L (g)	0.373 ± 0.021 (3)	0.287 ± 0.067 (3)	0.413 ± 0.294 (3)	0.307 ± 0.136 (3)
% Ovary_L (petcent)	0.00506 ± 0.00039 (3)	0.00413 ± 0.0011 (3)	0.0063 ± 0.00416 (3)	0.00439 ± 0.00157 (3)
Ovary_R (g)	0.367 ± 0.129 (3)	0.263 ± 0.061 (3)	0.437 ± 0.346 (3)	1.297 ± 1.706 (3)
% Ovary_R (percent)	0.00496 ± 0.00179 (3)	0.00376 ± 0.00086 (3)	0.00664 ± 0.00491 (3)	0.01856 ± 0.02442 (3)
Uterus&cervix (g)	8.987 ± 6.191 (3)	2.717 ± 0.881 (3)	6.143 ± 6.728 (3)	9.867 ± 6.315 (3)
% Uterus&cervix (percent)	0.11582 ± 0.07425 (3)	0.03964 ± 0.01557 (3)	0.09234 ± 0.09743 (3)	0.13934 ± 0.08506 (3)
Adrenal gland_L (g)	0.533 ± 0.07 (3)	0.53 ± 0.01 (3)	0.51 ± 0.082 (3)	0.55 ± 0.017 (3)
% Adrenal gland_L (petcent)	0.00729 ± 0.00161 (3)	0.00759 ± 0.00063 (3)	0.00792 ± 0.00085 (3)	0.00807 ± 0.00065 (3)
Adrenal gland_R (g)	0.507 ± 0.04 (3)	0.0493 ± 0.045 (3)	0.52 ± 0.13 (3)	0.517 ± 0.047 (3)
% Adrenal gland_R (percent)	0.00689 ± 0.00103 (3)	0.00709 ± 0.00108 (3)	0.00806 ± 0.00163 (3)	0.00757 ± 0.00065 (3)
Pituitary gland (g)	0.04 ± 0 (3)	0.0047 ± 0.0006 (3)	0.047 ± 0.006 (3)	0.047 ± 0.012 (3)
% Pituitary gland (percent)	0.00054 ± 0.00007 (3)	0.00067 ± 0.0001 (3)	0.00073 ± 0.00007 (3)	0.0007 ± 0.00025 (3)
Thymus	9.623 ± 0.826 (3)	7.01 ± 3 (3)	6.07 ± 1.172 (3)	6.89 ± 2.505 (3)
% Thymus (percent)	0.13063 ± 0.01642 (3)	0.09937 ± 0.04182 (3)	0.09419 ± 0.01293 (3)	0.0996 ± 0.032 (3)
Spleen (g)	35.437 ± 8.096 (3)	34.277 ± 14.514 (3)	23.107 ± 2.145 (3)	25.88 ± 6.568 (3)
% Spleen (percent)	0.48616 ± 0.13933 (3)	0.47894 ± 0.15798 (3)	0.36208 ± 0.05095 (3)	0.37442 ± 0.06781 (3)
Kidney_L (g)	15.707 ± 0.0656 (3)	15.783 ± 2.178 (3)	14.747 ± 1.132 (3)	13.897 ± 2.065 (3)
% Kidney_L (percent)	0.21372 ± 0.02802 (3)	0.22423 ± 0.01264 (3)	0.23005 ± 0.01595 (3)	0.20392 ± 0.03185 (3)
Kidney_R (g)	14.87 ± 1.125 (3)	15.337 ± 2.002 (3)	13.913 ± 0.501 (3)	14.16 ± 1.498 (3)
% Kidney_R (percent)	0.20247 ± 0.03137 (3)	0.218 ± 0.01153 (3)	0.21707 ± 0.0037 (3)	0.20786 ± 0.02574 (3)
Heart (g)	63.45 ± 2.323 (3)	62.22 ± 5.307 (3)	54.113 ± 5.804 (3)	58.553 ± 9.702 (3)
% Heart (percent)	0.86285 ± 0.10146 (3)	0.88972 ± 0.09 (3)	0.84358 ± 0.07857 (3)	0.85223 ± 0.06919 (3)
Lung (g)	67.99 ± 2.418 (3)	71.037 ± 6.289 (3)	61.443 ± 5.18 (3)	61.337 ± 5.678 (3)
% Lung (percent)	0.92385 ± 0.10217 (3)	1.01224 ± 0.02473 (3)	0.95916 ± 0.08628 (3)	0.89833 ± 0.07439 (3)
Brain (g)	67.92 ± 3.772 (3)	69.873 ± 1.988 (3)	70.433 ± 9.417 (3)	72.643 ± 4.912 (3)
% Brain (percent)	0.92267 ± 0.10241 (3)	0.99925 ± 0.06431 (3)	1.10516 ± 0.20042 (3)	1.06718 ± 0.11472 (3)
Liver&gall bladder (g)	217.127 ± 18.282 (3)	185.763 ± 43.925 (3)	175.863 ± 18.144 (3)	172.243 ± 13.737 (3)
% Liver&gall bladder (percent)	2.9468 ± 0.3611 (3)	2.34372 ± 0.5849 (3)	274,055 ± 0.22216 (3)	2.51995 ± 0.09763 (3)
Thyroid gland + PG_L (g)	0.35 ± 0.095 (3)	0.31 ± 0.089 (3)	0.333 ± 0.055 (3)	0.273 ± 0.045 (3)
% Thyroid gland + PG_L (percent)	0.00477 ± 0.00143 (3)	0.00446 ± 0.0014 (3)	0.00518 ± 0.0006 (3)	0.0004 ± 0.00062 (3)
Thyroid gland + PG_R (g)	0.323 ± 0.067 (3)	0.243 ± 0.076 (3)	0.343 ± 0.049 (3)	0.333 ± 0.1 (3)
% Thyroid gland + PG_R (percent)	0.00441 ± 0.0011 (3)	0.0035 ± 0.00116 (3)	0.00534 ± 0.00047 (3)	0.0049 ± 0.00154 (3)

Mean ± SD (Number of animals). L: left, R: right.

#### 3.3.9 Necropsy findings

Enlarged cerebral and partial discoloration of the lungs were found in 1 male in the control group. Some cysts in the pituitary gland and small left thyroid gland were found in 1 male in group 2000 mg/kg/day. Ovarian cyst formation was found in 1 female in group 2000 mg/kg/day. Abnormal shape and discoloration of the spleen were observed in one female in the control group, and uterine angular and vaginal thickening were observed in 2 females in the control group, and 1 females in group 1000 mg/kg/day and two cases were observed in group 2000 mg/kg/day.

In the recovery group, cerebral enlargement was found in 1 male in group 2000 mg/kg/day, and thickening of the uterine horn and vagina was found in 1 female in the control and in group 2000 mg/kg/day, respectively.

#### 3.3.10 Histopathological examination

Compare to the control group, male and female beagle dogs in 1000 mg/kg/day group showed few significant changes such as infiltration of inflammatory cell in the intestinal tract. Female beagle dogs in group 2000 mg/kg/day hypertrophy/hyperplasia of Kupffer cell was observed. Female and male beagle dogs in 500 mg/kg/day or more showed dilation of a sinusoid in the livers (Table 6, 7). Infiltration severity of inflammatory cells in the intestinal tract was increased in females. In addition, natural lesions in beagle dogs of certain age were observed.

**Table 6:** Histopathologic findings affected by the test article in the intestine and liver in male Beagle Dogs.

Sex	Male					
Groups	G1	G2	G3	G4	G1R	G4R
Number examined ORGAN/ FINDINGS	3	3	3	3	2	2
CECUM						
Infiltrate, Inflammatory Cell	0	0	1	2	1	2
Minimal (+1)	0	0	1	2	1	2
COLON						
Infiltrate, Inflammatory Cell	1	1	3	1	1	2
Minimal (+1)	1	1	3	1	1	2
DUODENUM						
Infiltrate, Inflammatory Cell	2	1	2	3	1	1
Minimal (+1)	2	0	2	1	0	1
Mild (+2)	0	1	0	2	1	0
ILEUM						
Infiltrate, Inflammatory Cell	3	2	3	2	2	2
Minimal (+1)	3	2	0	0	0	0
Mild (+2)	0	0	3	1	1	2
Moderate (+3)	0	0	0	1	1	0
JEJUNUM						
Infiltrate, Inflammatory Cell	3	0	3	2	2	1
Minimal (+1)	2	0	3	2	1	0
Mild (+2)	1	0	0	0	1	1
RECTUM						
Infiltrate, Inflammatory Cell	0	1	2	1	1	1
Minimal (+1)	0	1	2	1	1	1
LIVER						
Dilation, Sinusoid	3	3	2	3	1	1
Minimal (+1)	2	1	2	0	1	0
Mild (+2)	1	2	0	3	0	1

**Table 7:** Histopathologic findings affected by the test article in the intestine and liver in female Beagle Dogs.

Sex	Female					
Groups	G1	G2	G3	G4	G1R	G4R
Number examined Organ/Findings	3	3	3	3	2	2
CECUM						
Infiltrate, Inflammatory Cell	0	1	0	3	1	1
Minimal (+1)	0	1	0	2	1	1
Mild (+2)	0	0	0	1	0	0
COLON						
Infiltrate, Inflammatory Cell	1	2	2	3	2	1
Minimal (+1)	1	2	1	0	1	0
Mild (+2)	0	0	1	1	0	1
Moderate (+3)	0	0	0	2	1	0
DUODENUM						
Infiltrate, Inflammatory Cell	3	3	3	3	1	2
Minimal (+1)	2	3	0	0	0	1
Mild (+2)	1	0	2	0	1	0
Moderate (+3)	0	0	1	1	0	1
Severe (+4)	0	0	0	2	0	0
ILEUM						
Infiltrate, Inflammatory Cell	2	3	3	3	2	2
Minimal (+1)	0	3	2	0	1	1
Mild (+2)	1	0	0	0	1	0
Moderate (+3)	1	0	0	0	0	1
Severe (+4)	0	0	1	3	0	0
JEJUNUM						
Infiltrate, Inflammatory Cell	3	1	3	3	2	2
Minimal (+1)	1	1	1	0	1	1
Mild (+2)	2	0	2	0	1	1
Moderate (+3)	0	0	0	1	0	0
Severe (+4)	0	0	0	2	0	0
RECTUM						
Infiltrate, Inflammatory Cell	1	3	3	3	1	1
Minimal (+1)	1	3	2	0	1	1
Mild (+2)	0	0	1	2	0	0
Moderate (+3)	0	0	0	1	0	0
LIVER						
Hypertrophy/hyperplasia, Kupffer Cell	0	0	0	1	0	2
Minimal (+1)	0	0	0	0	0	1
Moderate (+3)	0	0	0	1	0	1
Dilation, Sinusoid	3	3	3	3	2	2
Minimal (+1)	3	0	0	0	1	0
Mild (+2)	0	3	1	0	0	1
Moderate (+3)	0	0	2	2	1	1
Severe (+4)	0	0	0	1	0	0

**Table 8:** Four-weeks repeated dose toxicity: absolute and relative organ weights of male dogs (main study) and absolute and relative organ weights of female dogs (main study).

Summary of absolute organ weights						
	Group (mg/kg/day) sex: male					
		G1 (0)	G2 (250)	G3 (500)	G4 (1000)	G5 (2000)
Ternminal BW (kg)	Mean	8.020	8.440	7.140	8.000	7.160
Lung (g)	Mean	76.550	80.220	57.050	86.100	64.660
%Lung (%)	Mean	0.95449	0.95047	0.79902	1.07625	0.90307
Heart (g)	Mean	55.290	59.100	42.920	67.580	55.340
%Heart (%)	Mean	0.68940	0.70024	0.60112	0.84475	0.77291
Brain (g)	Mean	72.920	74.240	66.440	77.260	79.550
%Brain (%)	Mean	0.90923	0.87962	0.93053	0.96575	1.11103
Liver&gall bladder (g)	Mean	217.860	272.780	235.530	262.610	191.710
%Liver&gall bladder (%)	Mean	2.71646	3.23199	3.29874	3.28263	2.67751
Spleen (g)	Mean	20.410	27.360	40.440	33.130	44.440
%Spleen (%)	Mean	0.25449	0.32417	0.56639	0.41413	0.62067
Kidney L (g)	Mean	20.620	17.720	16.630	20.140	15.430
% Kidney L (g)	Mean	0.25711	0.20995	0.23291	0.25175	0.21550
Kidney R (g)	Mean	20.640	17.430	16.010	20.200	15.270
% Kidney R (g)	Mean	0.25736	0.20652	0.22423	0.25250	0.21327
Thymus (g)	Mean	7.190	7.460	7.990	6.610	7.890
%Thymus (g)	Mean	0.08965	0.08839	0.11190	0.08263	0.11020

Summary of absolute organ weights						
	Group (mg/kg/day) sex: female					
		G1 (0)	G2 (250)	G3 (500)	G4 (1000)	G5 (2000)
Ternminal BW (kg)	Mean	6.660	6.860	7.620	6.260	7.120
Lung (g)	Mean	73.060	64.220	69.160	69.190	77.550
%Lung (%)	Mean	1.09700	0.93615	0.90761	1.10527	1.08919
Heart (g)	Mean	55.140	44.560	47.180	45.830	52.110
%Heart (%)	Mean	0.82793	0.64956	0.61916	0.73211	0.73188
Brain (g)	Mean	68.620	70.180	66.220	77.420	71.960
%Brain (%)	Mean	1.03033	1.02303	0.86903	1.23674	1.01067
Liver&gall bladder (g)	Mean	242.170	183.460	241.890	182.870	185.900
%Liver&gall bladder (%)	Mean	3.63619	2.67434	3.17441	2.92125	2.61096
Spleen (g)	Mean	40.630	44.640	48.570	29.510	32.620
%Spleen (%)	Mean	0.61006	0.65073	0.63740	0.47141	0.45815
Kidney L (g)	Mean	17.570	14.200	20.360	12.800	16.470
% Kidney L (g)	Mean	0.26381	0.20700	0.26719	0.20447	0.23132
Kidney R (g)	Mean	16.720	15.150	19.800	13.180	16.960
% Kidney R (g)	Mean	0.25105	0.22085	0.25984	0.21054	0.23820
Thymus (g)	Mean	6.770	5.060	7.710	6.920	5.400
%Thymus (g)	Mean	0.10165	0.07376	0.10118	0.11054	0.07584

## 4 Discussion

HAD-B1 focuses on lung cancer treatment and had proved its anti-lung cancer effects *in vitro* and *in vivo*, suppressing the progression of solid tumors in an A549 cell xenograft rat model [10]. HAD-B1 suppressed solid tumor growth compared to the vehicle or cisplatin treated control group in an A549 cell xenograft rat model and specific changes in body weight and blood biochemical parameters were not observed (ALT, AST, BUN, CRE, ALP). Therefore, it was not considered to have any toxicity.

The primary purpose of safety assessment in herbal medicine is to investigate the significance of adverse effects and determine the level of exposure at which the effects are ​observed. The objective of a nonclinical safety study consists of the identification of potential target organs for toxicity, the reversibility of toxicity, and the determination of NOAEL or NOEL [23]. The NOAEL is identified as the dose that has not caused significant adverse effects on animals in the specific nonclinical toxicology study [23].

In this present study, acute and sub-chronic toxicity study for HAD-B1 were performed to investigate toxicity profile for a potential toxicity to humans using the Beagle dog for non-rodent animal [24]. In acute toxicity study, there was no changes in body weight, significant clinical sign and mortality in any dose group. These results suggested that acute treatment of HAD-B1 does not affect normal growth of beagle dogs. The 4-week oral repeated DRF toxicity study was conducted to investigate general toxicity of repeated oral administration of HAD-B1 to beagle dogs and to determine dose levels for the subsequent 13-week repeated dose toxicity test. In the 4-week DRF study, no dead or moribund animals were observed during the experiment, and no toxicological changes were observed by HAD-B1 in general symptoms, body weight, feed intake, ophthalmic examination, electrocardiogram, urinalysis, blood and serum biochemical tests, and necropsy findings. Meanwhile, the absolute and relative weight of the spleen increased in males in the 500, 1000, 2000 mg/kg/day groups and decreased in females in the 1000 and 2000 mg/kg/day groups (Table 8). Based on this result, the high-dose which expected to be toxic to the experimental animals, was determine to be 2000 mg/kg/day and the 13-week repeated dose sub-chronic toxicity study was performed.

Since no toxicity in group 2000 mg/kg/day of HAD-B1 was observed in 4 weeks study, the groups of 13-week repeated dose toxicity and 4-week recovery studies was set at concentrations of 500, 1000, 2000 mg/kg/day to confirm the non-toxic doses, ascertain target organs and reversibility of HAD-B1. Food leftover, foamy vomiting, soft stool, excessive salivation, licking chops were dose-independent, noted occasionally in the control group, and lacked incidences. There abnormal observations could be naturally observed in experiments with beagle dogs. During the recovery period, decline of body weight gain in males at 2000 mg/kg/day was not observed during the treatment period. The average weight change was within the ChemOn Historical Control Data (HCD) and therefore, were not attributed to test substance. The mean of daily food consumption in females at 1000 and 2000 mg/kg/day showed a tendency to decrease with dose-response correlation, while no body weight change was observed which indicated HAD-B1 has no toxic effect. The significant reduction of QRS duration observed in female in group 2000 mg/kg/day during recovery period is due to relative result from no significant changes in major group, and high levels of the vehicle control group. It is determined that it is not affected by test substance.

The changes in urine volume in the male and female in group 1000 mg/kg/day on week 4 and 13 were not an effect of the test substance because the dose-response correlation had not been observed. The significant difference in HGB, HCT, and APTT observed in the hematological examination lacked dose-dependent responses to be under the test substance influence. The significant increase in RET in recovery group was determined not to be due to the test substance because no change was observed in the main group and there was no accompanying change in the red blood cell-related index. In clinical biochemical parameters, TCHO achieved statistical significance with dose-response correlation. However, since the index is within the normal range of the HCD, it is determined to have no toxicological effect. In addition, the significant changes in TG, BUN, Cl^−^, K^+^, CRE, and GLU observed at post-dosing were judged not treatment related because the dose-response correlation was not observed. Although the recovery group showed significant difference in A/G ratio, AST, CPK, K^+^ and Cl^−^ but the main group did not. They were within the normal range of HCD, hence considered not by the test article.

Significant organ weight changes of right testis, left thyroid and parathyroid gland (relative weight), stomach (absolute weight), right thyroid and parathyroid gland (absolute/relative weight), right adrenal (absolute weight) and pituitary (relative weight) observed in recovery group were not observed in major group. It is determined that the effect was by the test substance as they were within or slightly outside the normal range of the HCD.

All gross findings observed at necropsy were confirmed histopathological as the diagnosis was consistent with each finding. The result of histopathological examination shows inflammatory cell infiltration in the intestine, Kupffer cell hypertrophy/hyperplasia in the liver, and the dilation of a sinusoid in the liver of females dose-dependently. This can be translated that HAD-B1 is effective especially dose-responsively. Even if the dilation of sinusoids in males did now show dose-dependent results, it is assumed to be affected by the test substances based on the number of occurrences and the female’s test results. However, the invasion of inflammatory cells in the intestine is considered to be a response to the stimulation of orally administered test substance by monocyte in the mucous membrane, and it is not considered to be a toxicologically harmful response since it did not cause any damage to the intestinal tissue. Hypertrophy/hyperplasia of Kupffer cells in the liver is a result of phagocytosis of foreign material and estrogen treatment and is known to be found in inflammatory conditions caused by cytokines [25]. The dilation of sinusoid is a change in their lumen, that the local dilation is induced by administration of nitrosamine, pyrrolizidine alkaloids, or glucocorticoids, and hepatic microcirculation changes in hemodynamics are suggested as the mechanism of its occurrence [25]. Hypertrophy/hyperplasia of Kupffer cells in the liver in females and the dilation of sinusoid in males and females did not damage the parenchymal tissue of the liver, so it was not considered toxic. These changes have shown a tendency to recover during the four-week recovery period, but since they have not fully recovered, it would take longer to overcome.

Summarizing the results above, HAD-B1 administered orally to beagle dog at 500, 1000, 2000 mg/kg/day repeatedly for 13 weeks and a recovery period of 4 weeks was given, a decrease of mean daily feed consumption in females, infiltration of intestinal inflammatory cells in both sexes, and significant decrease in TCHO in females, Kupffer cell hypertrophy/hyperplasia in liver, and dilation of sinusoid were observed. However, they were not considered to be any toxic effect in the treated compared to control. Therefore, NOAEL of the HAD-B1 was determined to be 2000 mg/kg/day for both males and females, and no toxic target organ was observed. The results suggest that the oral administration of HAD-B1 at dose of 2000 mg/kg/d shows no toxic effects in Beagle dogs. The findings in this study provided a vital reference for selecting a safe application dosage of HAD-B1 for clinical use.

## 5 Conclusion

In conclusion, neither of oral administration of HAD-B1 on the acute toxicity study for beagle dogs nor repeated dose toxicity study for beagle dogs caused mortality, adverse effects, significant clinical signs, laboratory parameters, necropsy finding and abnormal results in histological examinations. Therefore, these results observed at the treatment doses (500, 1000, 2000 mg/kg BW) suggest that HAD-B1 does not produce any toxicological effects and is considered to be safe for clinical usage.

## CRediT authorship contribution statement

**Si-Yeon Song:** Conceptualization, Methodology, Writing – original draft. **Hyeok-Joon Chang:** Conceptualization, Methodology, Writing – original draft. **Soo-Dam Kim:** Writing – review & editing, Funding acquisition. **Eun-Bin Kwag:** Writing – review & editing. **So-Jung Park:** Conceptualization, Methodology, Writing – original draft. **Hwa-Seung Yoo:** Supervision, Project administration, Funding acquisition.

## Declaration of Competing Interest

The authors declare no conflicts of interest.
